# Supplementary material for: BORDER proteins protect expression of neighboring genes by promoting 3′ Pol II pausing in plants
Source: Nat Commun. 2019 Sep 25;10:4359. doi: 10.1038/s41467-019-12328-w (PMC6761125; doi:10.1038/s41467-019-12328-w)
Supplement: Supplementary file 1 — Supplementary Information [file 41467_2019_12328_MOESM1_ESM.pdf]

Supplementary Materials for

BORDER proteins protect expression of neighboring genes by promoting 3' Pol II pausing in plants

Xuhong Yu†, Pascal G.P. Martin†, and Scott D. Michaels

Correspondence to: michaels@indiana.edu
†: Equal contribution

This PDF file includes:
Supplementary Figures S1 to S9
Supplementary methods and detailed procedures for each supplementary figure
Supplementary references

```
BDR1    1 MSSNMGTELIDLETVKADSDAFGETNLMELVGSNDPPSLQHISVSEIEQEPMEISVSGPL
BDR2    1 ------------------------------------------------------------
BDR3    1 ----MSNNLLPQPCMQ-----MG-----QFINVP-TPIPELISN-------PEMRLSQPI

BDR1   61 SFQFEPEAVSFQSSMLVDTQSLMPQLQLPYSVERSVAA-CSNSVTGKRKSPPESTLSGSA
BDR2    1 -------------------------------MEMAEIN-GSMQLVGKHKSLPQTTLGGGS
BDR3   39 CSHISGGRQDF--------H-----VMLPSVVGLGSVNMDKTLLPGKRKSPLHPSVQ---

BDR1  120 TSEKLDASNKRVEPVHHRPWLEQFYSECIQR-----GHMPPPATLSTKTEHLPTPAKKVR
BDR2   29 ASEA--------PNKQVRPWLQQLSPASNGI-----LHIPTK-ILSQETLHSLMHGKKAT
BDR3   83 -------NKRMALPMEGRPWASAPMPVQLSSVSPRTQYLPASFVSKNSFVSFS-------

BDR1  175 QMEPASQKSGKQVMNKKQ-AGLSQGSVKTLNDGNESLRSKMKESLAAALALVHEHEESPK
BDR2   75 QTESAPQKPAKPVVNKKQHVPPPQRSVKAMEEVNESVRSKMRESLASALALVKKDDDSPK
BDR3  129 -------KPGKQAAARKPTLQ-KPMLLKPQSESSGSVRSKMRESLAGALAMVQCQMDVPN

BDR1  234 EKKNSETEEASVPVA---DSNEPASACGTSVTVGEDITPAMSTRDESFEQKNGNGRTLSQ
BDR2  135 GKENIGTVETPVITQENTQSFQPASPASISVPVGEGTMSEMPTSVESSVQKD-------S
BDR3  181 ESKMLDSETVANPLEGHV-SGPVSAASGVDVMVSNGSTEMLTLSDPSPVAG----ISV--

BDR1  291 ESSKDTKMNYVNQSDVQKTQFDEVFPCDDVRFSDSIFTGDELLQGNGLSWVLEPVSDFGE
BDR2  188 EIPVDIMMEDVIKFNVLKSQYDEVFPRDNVPFTDIIFPNDDLLHGNELSWDLEV-SDLGE
BDR3  234 QTVLPEILSIAKTSDAQVPEAVKPFVQDNVSYSDNVFSKDDLLQGNDLSWALESDIEFTV

BDR1  351 N-------------ETQKSFEDPELLASKIELELFKLFGGVNKKYKEKGRSLLFNLKDKN
BDR2  247 TKDY--------GTGGEKSFQDPKLLASKIEMELYKLFGGVNKKYRERGRSLLFNLKDKN
BDR3  294 NCQNEMIGAMANDGSLEKLLLDPQVLAFEIETELFKLFGGVNKKYKEKGRSLLFNLKDKS

BDR1  398 NPELRESVMSGKISPERLCNMTAEELASKELSQWRQAKAEEMAEMVVLRDTDIDVRNLVR
BDR2  299 NPELRERVMSEEISAERLCSMTAEELASKELSQWRQAKAEEMAKMVVLQDTDIDVRSLVR
BDR3  354 NPKLREKVMYGEIAAERLCSMSAEELASKELAEWRQAKAEEMAQMVVLQDTEVDIRSLVR

BDR1  458 KTHKGEFQVEIDPVDSGTVDVSAEITSNSKPRAKAKSSKSSTKATLKKNDSNDKNIKSNQ
BDR2  359 KTHKGEFQVEIEPVDRGTVDVSGGIMSRSKRRPRAKSHSVKT--ALKDEAAK------AD
BDR3  414 KTHKGEFQVEVEPMDSGSVEVSVGMSSINWSRTKNFKKKTP--SITKT-----------L

BDR1  518 GTSSAVTLPPTEEIDPMQGLSMDDEMKD-VGFLPPIVSLDEFMESLNSEPPFGSPHEHPP
BDR2  411 NEKSRSTPPSTEEIDPMQGLGIDDELKD-VEFLPPIVSLDEFMESLDSEPPFESPHGNSE
BDR3  461 GVK-NELNSSNESTGPINGVTIDDEMQAATGSLPPIVSLDEFMSSIDSESPSGFLSSDTE

BDR1  577 GKEDPASEKSDSKDGSHSKSPSRSPKQ----SPK------EPSESVSSKTELEKTNVISP
BDR2  470 MQVSP-SEKSDSEAGSDSKSPKGS--------PK------ELSDKSLPEAKPEKIDEVTP
BDR3  520 KKPSV-SDNNDVEE-VLVSSPKESANIDLCTSPVKAEALSPLTAKASSPVNAEDADIVSS

BDR1  627 KPDAGDQLDGDVSKPENTSLVDSIKEDRIWDGILQLSSASVVSVTGIFKSGEKAKTSEWP
BDR2  515 EFDANVKVDDDISRVEKAAALSDDKGERAWDGILQLSMSSVVPVAGIFKSGEKAETSEWP
BDR3  578 KPSSD--------LKSKTTSVFIPDGERLWEGVLQLSPSTVSSVIGILRSGEKTTTKEWP

BDR1  687 TMVEVKGRVRLSAFGKFVKELPLSRSRVLMVMNVVCKNGISQSQRDSLIEVAKSYVADQR
BDR2  575 AMVEVKGRVRLSGFGKFIQELPKSRTRALMVMYLAYKDGISESQRGSLIEVIDSYVADQR
BDR3  630 ILLEIKGRVRLDAFEKFVRELPNSRSRAVMVMCFVCKEECSKTEQENISEVVDSYAKDGR
```

```
BDR1   747 VGYAEPTSGVELYLCPTLGETLDLLSKIISKDYLDEVKCSEDIGLIGVVVWRRAVVASPG
BDR2   635 VGYAEPASGVELYLCPTRGETLDLLNKVISQEQLDEVKS-LDIGLVGVVVWRRAVVPKPG
BDR3   690 VGYAEPASGVELYLCPTRGRTVEILNKIVPRNQLDFLKSINDDGLIGVVVWRRPQFKKSP

BDR1   807 SRHKPGFKRQHSSTGTKRSVLAPENQKSRSVSVTNPSVVNVESMRNHGLVGCD-DDDEDM
BDR2   694 SGSKRQHSF-SSSIGSKTSVL-PVNKKQRVHVTEKPLVVASMRNHHHGYVKHDTAADDDV
BDR3   750 LSNNSHKN--HRE---KGS-----------------SLTTYNTSRYSNMLQVNNDDGDDV

BDR1   866 PPGFGPVA--AKDDDDLPEFNFNSSSGPVTSSPRPPLQSRSLDQVRELILKYGNSTGSGS
BDR2   752 PPGFGPVA--SRDEDDLPEFNFNSSVVPVSSPQPLPAQSKSLDQVRKLIHKYGKSAST--
BDR3   788 PPGFGPMTMARDEDDDLPEFNYFSSGDVVVNRT------SRSVSVRELIQKYGKSEPLRN

BDR1   924 KRPWDGHDDDDDDDIPEWQPQLPP-----PPPDLSPQFHSGTMARPPAQRPVAGPPSGWK
BDR2   808 -----YDDDDDEDDIPEWQPHVPSHQLPPPPPP-PLGFR------P---EVFRPPQDGWY
BDR3   842 Q----SYNDNDNDILPEWQPQSNWTLG-------VTHVNGGS---------MVRPCSEWW

BDR1   979 ANQNAPRQQQYSA--RRNRGF
BDR2   853 DNQNGGSGQHYERNQSRNRGF
BDR3   882 SHQDGRGGY------------
```

Supplementary Figure 1. Protein alignment of BDR proteins. Alignment generated by CLUSTAL O (1.2.4).

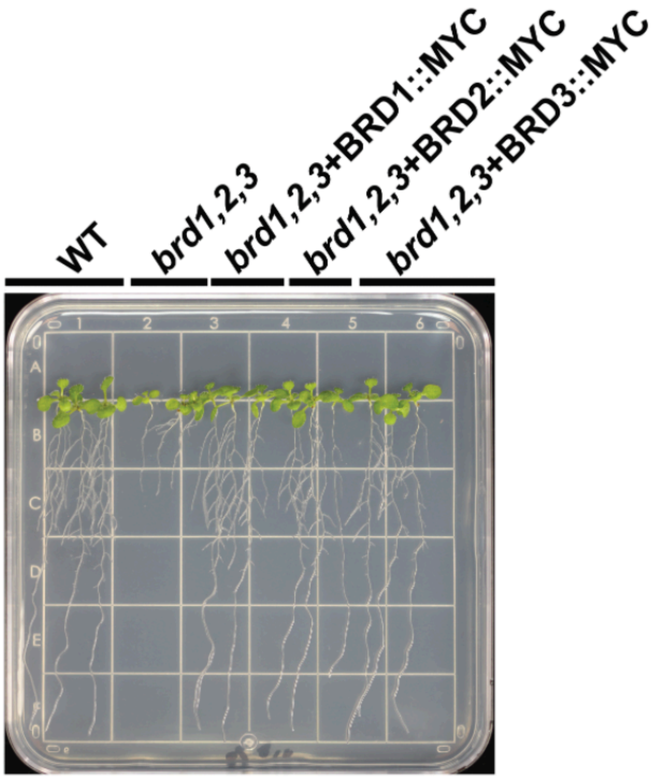

Supplementary Figure 2. Rescue of *bdr1,2,3* root growth using MYC-tagged BDR1, BDR2, and BDR3 constructs.

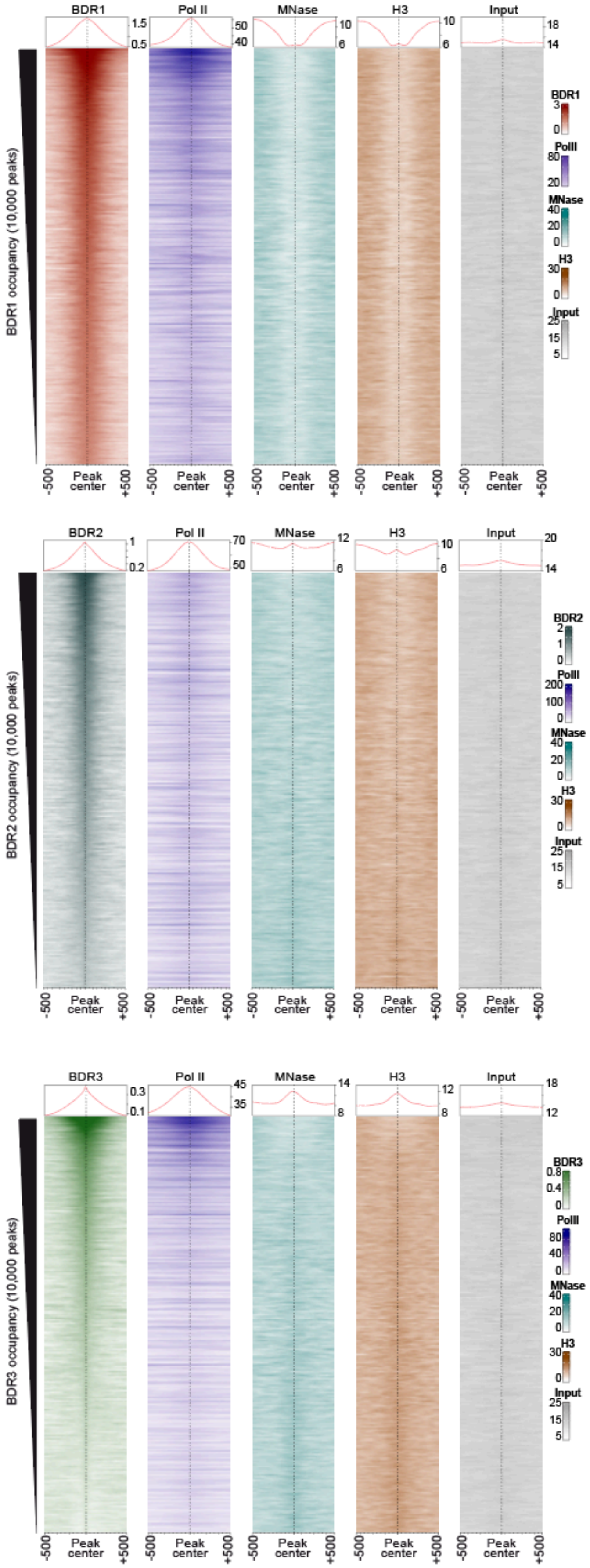

Supplementary Figure 3. BDR protein colocalize with Pol II. Correlation of BDR1, BDR2, and BDR3 occupancy and other genomic features. Heatmap and average profiles (top) of ChIP-seq or MNase-seq signals around BDR peaks sorted by levels of BDR occupancy; the top 10,000 regions are shown.

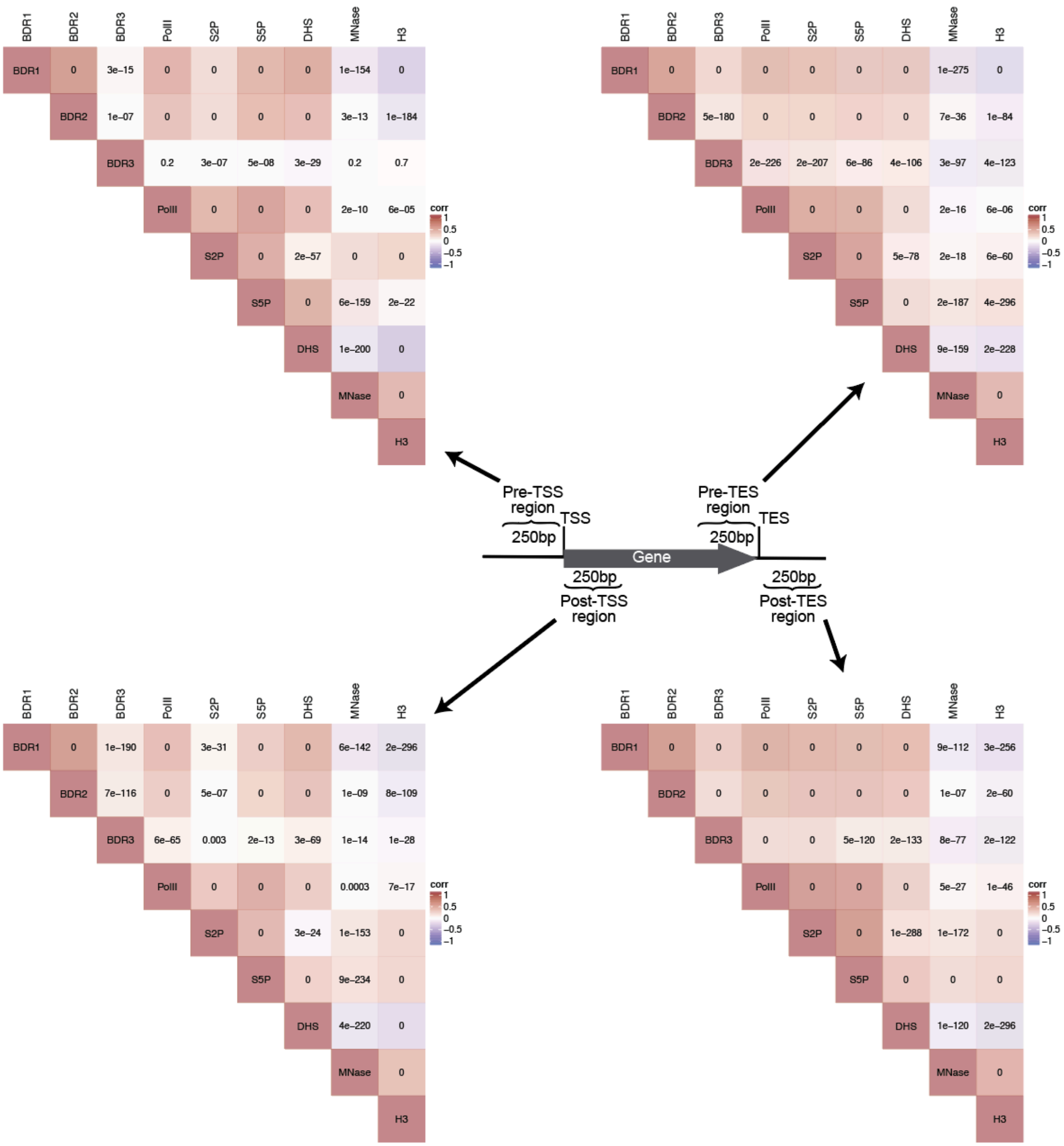

Supplementary Figure 4. Correlation between genomic features in 250bp regions immediately before the TSS, after the TSS, before the TES, and after the TES. Strength of the correlation is shown by color and corrected p values are shown. p values < 1e-300 are shown as 0.

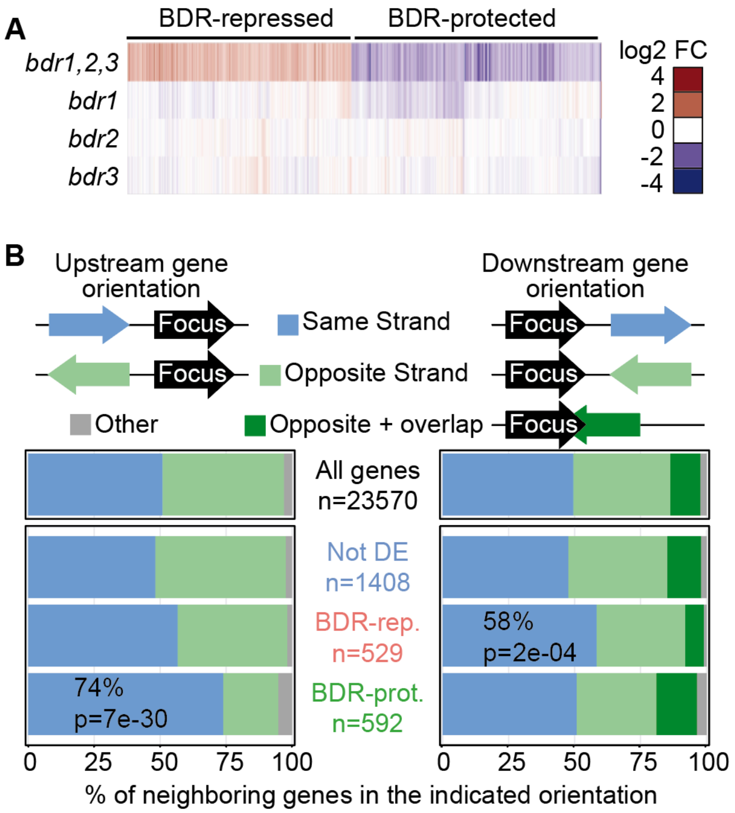

Supplementary Figure 5. BDR-protected genes occur in a specific genomic context.
A) Identification of BDR-protected (downregulated in *bdr1,2,3*) and BDR-repressed (upregulated in *bdr1,2,3*) genes by RNA-seq analysis (Supplementary Data 1, Table S2).
B) BDR-protected genes preferentially have an upstream gene on the same strand. Orientation of upstream and downstream neighbors of all expressed genes, non-differentially expressed control genes, or BDR-protected genes. Enrichment for a given orientation is evaluated by Fisher exact test with a BH p-value correction. Adjusted p-values below 0.01 are shown.

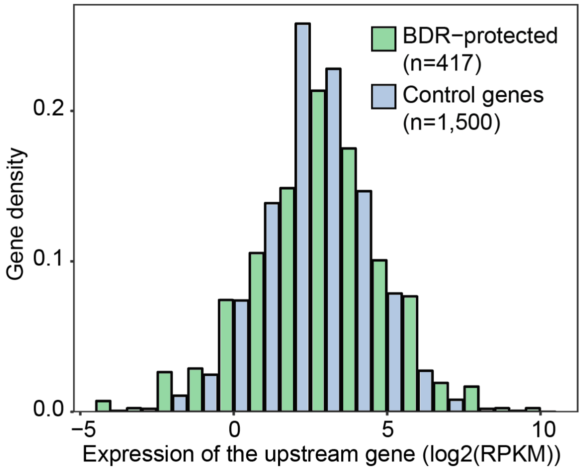

Supplementary Figure 6. Expression of a control set of 1,500 genes that were selected to have a similar expression distribution to the upstream neighbors of BDR-protected genes in Arabidopsis seedlings.

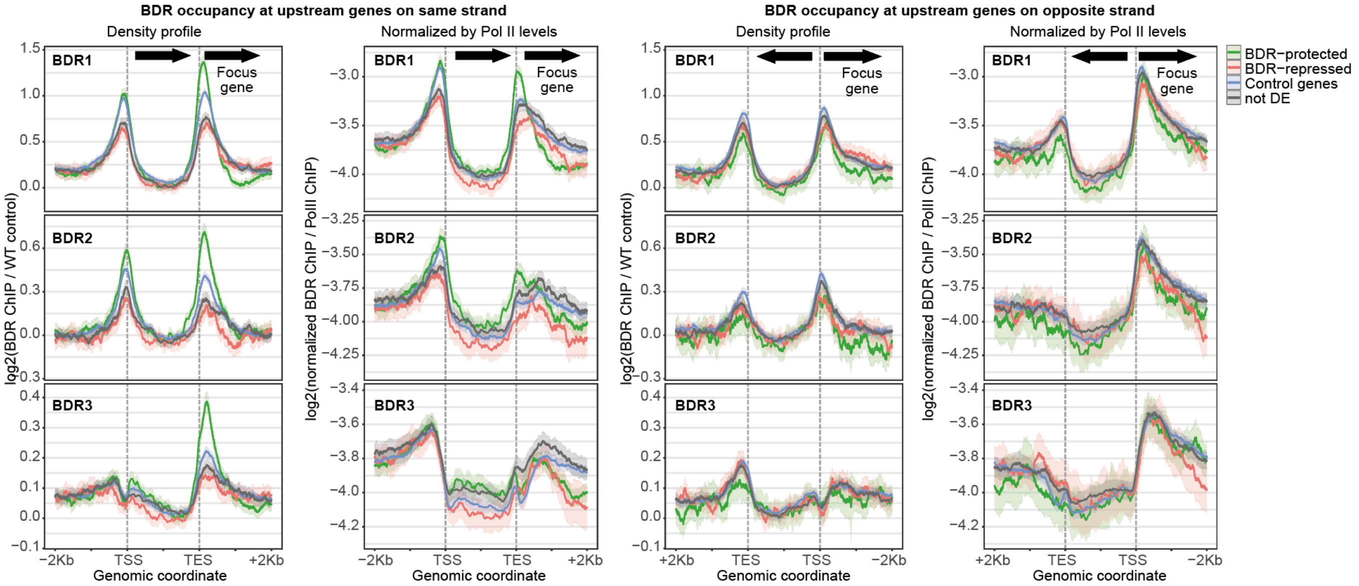

Supplementary Figure 7. BDR protein enrichment at the borders of the tandem upstream neighbors of BDR-protected genes.

Metagene profiles of BDR1 and BDR2 ChIP-seq coverage for the upstream neighbors of BDR-protected, BDR-repressed, expression-matched control genes, and non-differentially expressed genes. BDR ChIP-seq data is presented as wild-type-normalized read density and following normalization to Pol II.

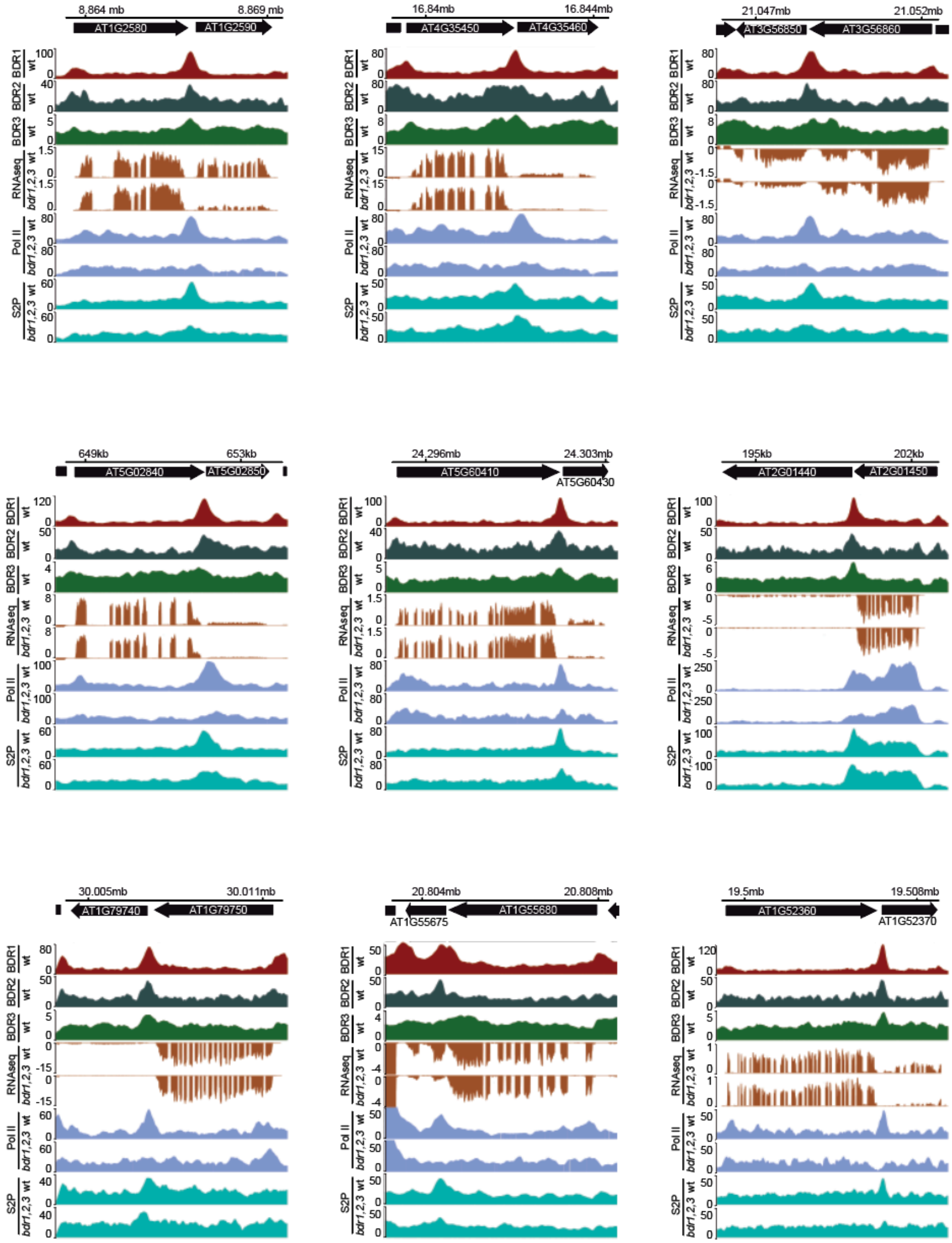

Supplementary Figure 8. Genome browser tracks showing BDR-protected genes and their upstream neighbors.

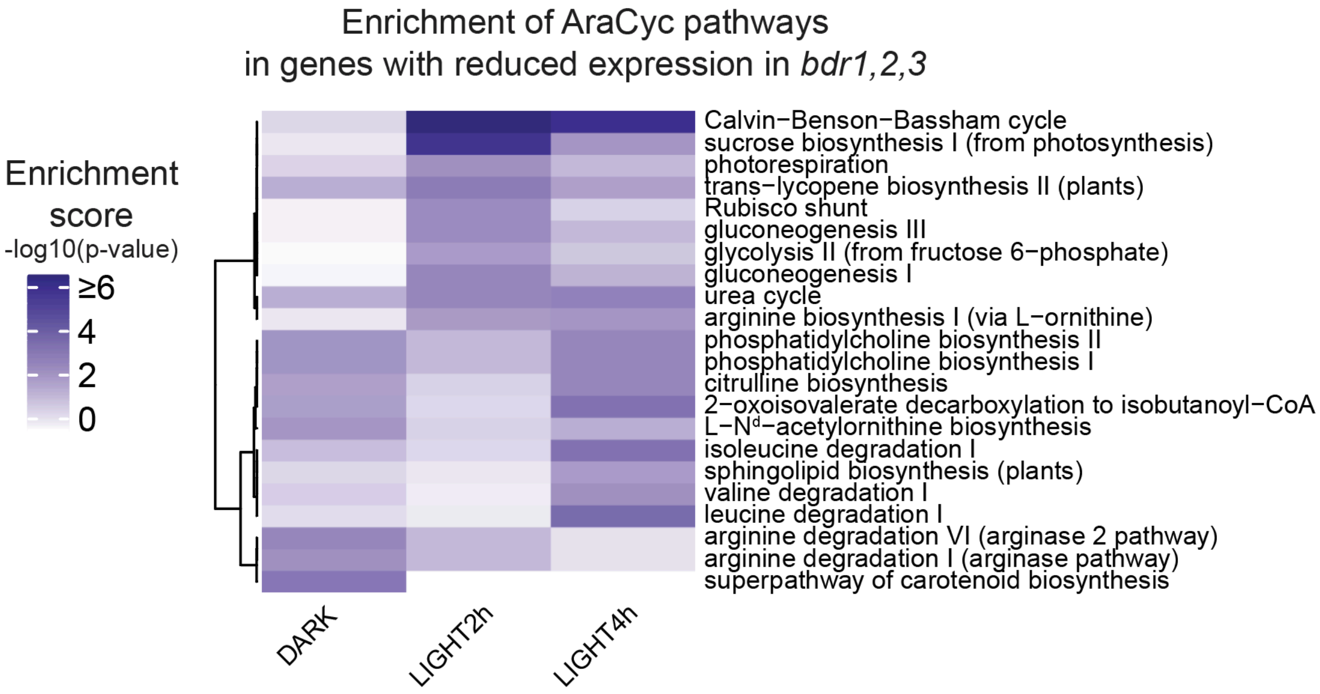

Supplementary Figure 9. Biochemical pathway analysis of genes showing reduced induction in *bdr1,2,3*.
For each condition (dark, light 2h or light 4h), we selected genes that showed significantly lower expression in *bdr1,2,3* compared to wild type. These genes were analyzed with the goseq Bioconductor package to determine the enrichment ($p<0.01$, at least 3 DE genes in the pathway) of AraCyc pathways (www.plantcyc.org). Enrichment scores were plotted as a heatmap (-log10(p-value) for all pathways that were significantly enriched in at least one condition. The rows/pathways were reordered by hierarchical clustering using Euclidean distance and Ward agglomeration criterion. This analysis shows that several genes in the Calvin-Benson-Bassham cycle display an altered induction in the *bdr1,2,3* mutant compared to wild-type plants.

Supplementary bioinformatic methods for each figure
Supplementary Figure 1. Protein sequences for BDR1, BDR2 and BDR3 were aligned using Clustal Omega version 1.2.4 [1] .

Supplementary Figure 3.
For each BDR::MYC ChIP-seq, we sorted the top 10,000 peaks by decreasing level of normalized coverage near the peak summit (+/-100bp around peak summit). We then represented as heatmaps the signal obtained around these peaks (+/-500bp) for the BDR ChIP-seq (GSE113059 for BDR1 and BDR2 or GSE131772for BDR3), for Pol II ChIP-seq (GSE113078), for MNase-seq and H3 ChIP-seq (GSE113076) as well as for input DNA from BDR ChIP-seq control (GSE113059).

Supplementary Figure 4. The signal of normalized BDR1, BDR2 and BDR3 ChIP-seq, PolII, S2P and S5P ChIP-seq, DNAse hypersensitivity (DHS, GSE34318), MNase-seq and H3 native ChIP-seq was extracted in each region for all expressed protein-coding genes (n=21,290) and the Spearman correlation coefficient was calculated (in order to account for possible non-linear relationships). The reported p-values are from a t-test evaluating if the correlations are significantly different from 0 and were corrected for multiplicity by the Benjamini-Hochberg procedure.

Supplementary Figure 5. Panel A. The heatmap was produced with the EnrichedHeatmap package [2] using as input log2(mutant/wild-type) obtained from DESeq2 analysis on a selection of 1124 genes that were significantly up- or downregulated (FDR<5%) in at least one of the mutant genotypes (Table S3).

Supplementary Figure 5. Panel B. For all expressed genes (defined by positive read counts in RNA-seq study GSE112441 and after removing genes located at chromosome borders; n=23570), for control "Not DE" genes (n=1408), and for genes upregulated (BDR-repressed, n=529) or downregulated (BDR-protected, n=592) in the *bdr1,2,3* triple mutant compared to wild-type plants, we counted the number of upstream genes located on the same strand (blue), on the opposite strand (green) or with an overlapping upstream gene (grey) and calculated the corresponding proportions. We did the same for the downstream gene neighbors, but we also individualized from the "Other" category the frequent situation of an overlapping gene on the opposite strand (dark green). Significance of the enrichment for a given orientation was assessed by a Fisher exact test with a Benjamini-Hochberg (BH) correction. Only adjusted p-values below 0.01 are shown.

Supplementary Figure 6. We sampled 1,500 genes from non-differentially expressed genes having an upstream gene neighbor on the same strand so that the expression distribution of their upstream genes follows a normal distribution with mean and variance identical to the upstream tandem genes of BDR-protected genes. The histograms represent the distribution of the expression levels of these upstream tandem gene neighbors for BDR-protected genes or the control gene set.

Supplementary Figure 7. We analyzed the occupancy of BDR1 (GSE113059), BDR2 (GSE113059) and BDR3 (GSE131772) at genes located upstream, either on the same strand (left plots) or on the opposite strand (right plots) for the following groups of genes: BDR-protected genes, (n=592), BDR repressed genes (n=529), “Not DE” control genes (n=1408) or expression level-matched controls (n=1500 for each orientation). For each orientation of the upstream gene, we plotted metagene profiles representing the ChIP-seq coverage of

BDR::MYC protein normalized by their corresponding wild-type control ChIP only (log2(BDR ChIP / WT control)) or also by Pol II (GSE113078) ChIP-seq coverage (log2(normalized BDR ChIP / Pol II ChIP)). Average normalized coverages (solid lines) and 95% confidence intervals (shades) are represented.

Supplementary Figure 8. Coverages from ChIP-seq fragments of BDR1::MYC (GSE113059), BDR2::MYC (GSE113059), BDR3::MYC (GSE131772), Pol II (GSE113078) and Pol II S2P (GSE113075) in wild-type and *bdr1,2,3* triple mutant (units: FP10M) and average coverage from RNA-seq (GSE112441) fragments obtained from 3 wild-type or *bdr1,2,3* mutant samples (units: RPM, sign indicating on which strand the reads align) were plotted with the Gviz R package [3] for genomic regions corresponding to 9 BDR-protected genes and their upstream gene neighbor on the same strand.

Supplementary Figure 9. Using the RNA-seq data GSE112442, we identified all genes downregulated in bdr1,2,3 mutant compared to wild-type under the dark, light 2h or light 4h conditions (DESeq2, FDR<5%). Using Bioconductor goseq package [4] we identified all Aracyc pathways (www.plantcyc.org) that were significantly enriched in at least one of these gene sets (p<0.01 and at least 3 differentially expressed genes in the pathway) and plotted the corresponding –log10(p-value) as a heatmap in which rows were re-organized by hierarchical ascending clustering using the Euclidean distance and Ward agglomeration criterion. To limit the effect of extremely low p-values in the heatmap we set the maximum color intensity at p-value=1e-06.

Supplementary References

1. Sievers, F. et al. Fast, scalable generation of high-quality protein multiple sequence alignments using Clustal Omega. *Mol Syst Biol* **7**, 539 (2011).
2. Gu, Z., Eils, R., Schlesner, M. & Ishaque, N. EnrichedHeatmap: an R/Bioconductor package for comprehensive visualization of genomic signal associations. *BMC Genomics* **19**, 234 (2018).
3. Hahne F, I. Visualizing Genomic Data Using Gviz and Bioconductor. in *Statistical Genomics: Methods and Protocols* (eds. E, M. & S, D.) (Springer New York, New York, 2016).
4. Young, M.D., Wakefield, M.J., Smyth, G.K. & Oshlack, A. Gene ontology analysis for RNA-seq: accounting for selection bias. *Genome Biol* **11**, R14 (2010).
