# Supplementary material for: BORDER proteins protect expression of neighboring genes by promoting 3′ Pol II pausing in plants
Source: Nat Commun. 2019 Sep 25;10:4359. doi: 10.1038/s41467-019-12328-w (PMC6761125; doi:10.1038/s41467-019-12328-w)
Supplement: Supplementary file 3 — Description of Additional Supplementary Files [file 41467_2019_12328_MOESM3_ESM.pdf]

Description of Additional Supplementary Files

File Name: Supplementary Data 1

Description: 1) Raw data from root-growth assays (Fig. 1B,C).

2) Binning of Arabidopsis genes by expression level (Fig. 2B).

3) Differentially expressed genes between wild type and *bdr* mutants (Supplementary Fig. 5A).

File Name: Supplementary Data 2

Description: Blacklist of genomic regions with systematically high signal in ChIP-seq control samples.
