# Supplementary material for: BORDER proteins protect expression of neighboring genes by promoting 3′ Pol II pausing in plants
Source: Nat Commun. 2019 Sep 25;10:4359. doi: 10.1038/s41467-019-12328-w (PMC6761125; doi:10.1038/s41467-019-12328-w)
Supplement: Supplementary file 6 — Reporting Summary [file 41467_2019_12328_MOESM6_ESM.pdf]

nature research

Corresponding author(s): *Double-blind peer review submissions: write DBPR and your manuscript number here instead of author names.*

Last updated by author(s): *YYYY-MM-DD*

Reporting Summary

Nature Research wishes to improve the reproducibility of the work that we publish. This form provides structure for consistency and transparency in reporting. For further information on Nature Research policies, see Authors & Referees and the Editorial Policy Checklist.

Statistics

For all statistical analyses, confirm that the following items are present in the figure legend, table legend, main text, or Methods section.

n/a	Confirmed	
☐	☒	The exact sample size (n) for each experimental group/condition, given as a discrete number and unit of measurement
☐	☒	A statement on whether measurements were taken from distinct samples or whether the same sample was measured repeatedly
☐	☒	The statistical test(s) used AND whether they are one- or two-sided<br>*Only common tests should be described solely by name; describe more complex techniques in the Methods section.*
☐	☒	A description of all covariates tested
☐	☒	A description of any assumptions or corrections, such as tests of normality and adjustment for multiple comparisons
☐	☒	A full description of the statistical parameters including central tendency (e.g. means) or other basic estimates (e.g. regression coefficient) AND variation (e.g. standard deviation) or associated estimates of uncertainty (e.g. confidence intervals)
☐	☒	For null hypothesis testing, the test statistic (e.g. F, t, r) with confidence intervals, effect sizes, degrees of freedom and P value noted<br>*Give P values as exact values whenever suitable.*
☒	☐	For Bayesian analysis, information on the choice of priors and Markov chain Monte Carlo settings
☒	☐	For hierarchical and complex designs, identification of the appropriate level for tests and full reporting of outcomes
☐	☒	Estimates of effect sizes (e.g. Cohen's d, Pearson's r), indicating how they were calculated

Our web collection on statistics for biologists contains articles on many of the points above.

Software and code

Policy information about availability of computer code

Data collection	No software was used
Data analysis	Unless otherwise stated, all analysis were done under R v3.3.2 using mainly packages from Bioconductor v 3.4.<br>Original code used for the analysis of gene neighbors is being further developed in the GeneNeighborhood R package available at https://github.com/pgpmartin/GeneNeighborhood.<br>Detailed procedures for the production of each figure are provided in Methods

For manuscripts utilizing custom algorithms or software that are central to the research but not yet described in published literature, software must be made available to editors/reviewers. We strongly encourage code deposition in a community repository (e.g. GitHub). See the Nature Research guidelines for submitting code & software for further information.

Data

Policy information about availability of data

All manuscripts must include a data availability statement. This statement should provide the following information, where applicable:

- Accession codes, unique identifiers, or web links for publicly available datasets
- A list of figures that have associated raw data
- A description of any restrictions on data availability

All raw and processed sequencing data, as well as details of the experimental procedures and data analysis methods are provided in GEO SuperSeries GSE112443

Field-specific reporting

Please select the one below that is the best fit for your research. If you are not sure, read the appropriate sections before making your selection.

☒ Life sciences ☐ Behavioural & social sciences ☐ Ecological, evolutionary & environmental sciences

For a reference copy of the document with all sections, see nature.com/documents/nr-reporting-summary-flat.pdf

Life sciences study design

All studies must disclose on these points even when the disclosure is negative.

Sample size	We did not perform sample-size calculations. Sample sizes for sequencing studies were essentially maximized based on available funding, expected sequencing depth and experimental capacity (e.g. ability to handle large number of tubes simultaneously in ChIP-seq experiments)
Data exclusions	As stated in GEO entry GSE112440, sample WT_12 was removed from the diffenrential expression analysis due to its low sequencing depth compared to the other samples.
Replication	The RNA-seq study to identify genes differentially expressed between bdr1,2,3 triple mutant and wild-type plants was entirely replicated (2 independent studies: GSE112440 and GSE112441). Both studies involved biological triplicates in each group (except after removal of WT_12 in GSE112440, see above).<br>Replicates for ChIP-seq are described below. ChIP-seq on RNA polymerase II were performed in duplicates (or triplicates for RNAPII ChIP), using 3 different antibodies targeting different phosphorylation states of the largest subunit (RNAPII, S2P and S5P).
Randomization	Samples/individuals were randomly allocated to the treatment groups (MPA, 6AU or light experiments)
Blinding	Except for the personnel of the sequencing facility, investigators were not blinded to group allocation during data collection or analysis.

Reporting for specific materials, systems and methods

We require information from authors about some types of materials, experimental systems and methods used in many studies. Here, indicate whether each material, system or method listed is relevant to your study. If you are not sure if a list item applies to your research, read the appropriate section before selecting a response.

Materials & experimental systems

n/a	Involved in the study	
☐	☒	Antibodies
☐	☐	Eukaryotic cell lines
☐	☐	Palaeontology
☐	☐	Animals and other organisms
☐	☐	Human research participants
☐	☐	Clinical data

Methods

n/a	Involved in the study	
☐	☒	ChIP-seq
☐	☐	Flow cytometry
☐	☐	MRI-based neuroimaging

Antibodies

Antibodies used	Antibodies are described (including lot numbers) for each sample in GEO entry GEO112443.<br>Anti-RNA polymerase II CTD repeat YSPTSPS antibody [8WG16] - ChIP Grade (abcam, ab817, lot #: GR261055-1, GR261055-2 or GR299078-3)<br>Anti-RNA polymerase II CTD repeat YSPTSPS (phospho S2) antibody - ChIP Grade (abcam, ab5095, lot #: GR270578-1 or GR295145-1)<br>Anti-RNA polymerase II CTD repeat YSPTSPS (phospho S5) antibody - ChIP Grade (abcam, ab513, lot #: GR118198-2 or GR278215-1)<br>Anti-Histone H3 antibody - Nuclear Loading Control and ChIP Grade (abcam, ab1791, lot #: GR312884-1)<br>Mouse IgG2a [MOPC-173] Isotype Control- ChIP Grade (abcam, ab18413, lot # GR249563-7)<br>Anti-Myc Tag Antibody, clone 4A6 (Millipore-sigma, 05-724, lot #: 2720363).
Validation	All the antibodies used in this study are commercially available and validated by their corresponding manufacturers.

Eukaryotic cell lines

Policy information about cell lines

Cell line source(s)	*State the source of each cell line used.*

Authentication	*Describe the authentication procedures for each cell line used OR declare that none of the cell lines used were authenticated.*
Mycoplasma contamination	*Confirm that all cell lines tested negative for mycoplasma contamination OR describe the results of the testing for mycoplasma contamination OR declare that the cell lines were not tested for mycoplasma contamination.*
Commonly misidentified lines (See ICLAC register)	*Name any commonly misidentified cell lines used in the study and provide a rationale for their use.*

Palaeontology

Specimen provenance	*Provide provenance information for specimens and describe permits that were obtained for the work (including the name of the issuing authority, the date of issue, and any identifying information).*
Specimen deposition	*Indicate where the specimens have been deposited to permit free access by other researchers.*
Dating methods	*If new dates are provided, describe how they were obtained (e.g. collection, storage, sample pretreatment and measurement), where they were obtained (i.e. lab name), the calibration program and the protocol for quality assurance OR state that no new dates are provided.*

☐ Tick this box to confirm that the raw and calibrated dates are available in the paper or in Supplementary Information.

Animals and other organisms

Policy information about studies involving animals; ARRIVE guidelines recommended for reporting animal research

Laboratory animals	*For laboratory animals, report species, strain, sex and age OR state that the study did not involve laboratory animals.*
Wild animals	*Provide details on animals observed in or captured in the field; report species, sex and age where possible. Describe how animals were caught and transported and what happened to captive animals after the study (if killed, explain why and describe method; if released, say where and when) OR state that the study did not involve wild animals.*
Field-collected samples	*For laboratory work with field-collected samples, describe all relevant parameters such as housing, maintenance, temperature, photoperiod and end-of-experiment protocol OR state that the study did not involve samples collected from the field.*
Ethics oversight	*Identify the organization(s) that approved or provided guidance on the study protocol, OR state that no ethical approval or guidance was required and explain why not.*

Note that full information on the approval of the study protocol must also be provided in the manuscript.

Human research participants

Policy information about studies involving human research participants

Population characteristics	*Describe the covariate-relevant population characteristics of the human research participants (e.g. age, gender, genotypic information, past and current diagnosis and treatment categories). If you filled out the behavioural & social sciences study design questions and have nothing to add here, write "See above."*
Recruitment	*Describe how participants were recruited. Outline any potential self-selection bias or other biases that may be present and how these are likely to impact results.*
Ethics oversight	*Identify the organization(s) that approved the study protocol.*

Note that full information on the approval of the study protocol must also be provided in the manuscript.

Clinical data

Policy information about clinical studies

All manuscripts should comply with the ICMJE guidelines for publication of clinical research and a completed CONSORT checklist must be included with all submissions.

Clinical trial registration	*Provide the trial registration number from ClinicalTrials.gov or an equivalent agency.*
Study protocol	*Note where the full trial protocol can be accessed OR if not available, explain why.*
Data collection	*Describe the settings and locales of data collection, noting the time periods of recruitment and data collection.*
Outcomes	*Describe how you pre-defined primary and secondary outcome measures and how you assessed these measures.*

ChIP-seq

Data deposition

☒ Confirm that both raw and final processed data have been deposited in a public database such as GEO.

☒ Confirm that you have deposited or provided access to graph files (e.g. BED files) for the called peaks.

Data access links<br>*May remain private before publication.*	To review GEO accession GSE112443:<br>go to https://www.ncbi.nlm.nih.gov/geo/query/acc.cgi?acc=GSE112443
Files in database submission	ChIP-seq files related to this study are in GEO entries GSE113059 (Myc-BDR1 and Myc-BDR2 ChIP-seq) and GSE113078 (RNA polymerase II ChIP-seq). Both Series are part of GSE112443 SuperSeries.<br>Fastq, bigwig andbed files (BDR1 and BDR2 peaks) are provided for the following samples in GSE113059:<br>- Myc_BDR1<br>- Myc_BDR2<br>- Myc_WT<br>- input_BDR2_lib1_seq1<br>- input_BDR2_lib1_seq2<br>- input_BDR2_lib2<br>- input_BDR1<br>- input_WT<br>Fastq and bigwig files are provided for the following samples in GSE113078:<br>- RNAPII_WT_rep1<br>- RNAPII_bdrs_rep1<br>- S2P_WT_rep1<br>- S2P_bdrs_rep1<br>- S5P_WT_rep1<br>- S5P_bdrs_rep1<br>- input_WT_rep1_lib1<br>- input_bdrs_rep1_lib1<br>- RNAPII_WT_rep2_seq1<br>- RNAPII_bdrs_rep2_seq1<br>- S2P_WT_rep2<br>- S2P_bdrs_rep2<br>- S5P_WT_rep2<br>- S5P_bdrs_rep2<br>- RNAPII_WT_rep2_seq2<br>- RNAPII_bdrs_rep2_seq2<br>- input_WT_rep1_seq2<br>- RNAPII_bdrs_rep2_seq2<br>- input_WT_rep1_lib2<br>- input_bdrs_rep1_lib2<br>- IgG_WT_rep3<br>- input_WT_rep3<br>- RNAPII_WT_rep3
Genome browser session<br>(e.g. UCSC)	http://jbrowseiu.pgpmartin.fr

Methodology

Replicates	In the file names above _rep1, 2 or 3 designates independent biological replicates. _seq1 or 2 indicates independent sequencing of the same library and _lib1 or 2 designates independent technical replicates at the level of library preparation (and sequencing). The results presented here were supported by all replictates.
Sequencing depth	*Describe the sequencing depth for each experiment, providing the total number of reads, uniquely mapped reads, length of reads and whether they were paired- or single-end.*
Antibodies	Antibodies used for ChIP-seq are described in the Antibody section above and for each sample in GEO entry GEO112443 (including lot numbers).
Peak calling parameters	Reads were mapped to the TAIR10 genome using Bowtie2 (v2.2.6, Langmead & Salzberg, Nature Methods, 2012) with the --dovetail parameter and maximum insert size of 1kb<br>Peaks for BDR1 and BDR2 were called using MACS2 (v2.1.0, Zhang et al., Genome Biology, 2008) in paired-end mode using Myc_WT as control. Peaks with a p-value<0.01 were kept. Peaks located in blacklisted regions were removed (see blacklist bed file provided as supplemental file).
Data quality	Read quality was evaluated with fastqc (v 0.11.14)<br>Adapters were trimmed with Trimmomatic (v0.33, Bolger, Lohse & Usabel, Bioinformatics, 2014) in paired end mode<br>After alignment, duplicate reads were identified with Picard (v2.2.4) MarkDuplicates and removed. Samtools v 1.3 was used to keep only reads mapped in proper pairs with mapping quality (MapQ) above 2.

Software	In addition to the software above, ChIP-seq data were analyzed with custom R scripts under R (v3.3.2) and Bioconductor (v3.4)

Flow Cytometry

Plots

Confirm that:

- [ ] The axis labels state the marker and fluorochrome used (e.g. CD4-FITC).
- [ ] The axis scales are clearly visible. Include numbers along axes only for bottom left plot of group (a 'group' is an analysis of identical markers).
- [ ] All plots are contour plots with outliers or pseudocolor plots.
- [ ] A numerical value for number of cells or percentage (with statistics) is provided.

Methodology

Sample preparation	*Describe the sample preparation, detailing the biological source of the cells and any tissue processing steps used.*
Instrument	*Identify the instrument used for data collection, specifying make and model number.*
Software	*Describe the software used to collect and analyze the flow cytometry data. For custom code that has been deposited into a community repository, provide accession details.*
Cell population abundance	*Describe the abundance of the relevant cell populations within post-sort fractions, providing details on the purity of the samples and how it was determined.*
Gating strategy	*Describe the gating strategy used for all relevant experiments, specifying the preliminary FSC/SSC gates of the starting cell population, indicating where boundaries between "positive" and "negative" staining cell populations are defined.*

- [ ] Tick this box to confirm that a figure exemplifying the gating strategy is provided in the Supplementary Information.

Magnetic resonance imaging

Experimental design

Design type	*Indicate task or resting state; event-related or block design.*
Design specifications	*Specify the number of blocks, trials or experimental units per session and/or subject, and specify the length of each trial or block (if trials are blocked) and interval between trials.*
Behavioral performance measures	*State number and/or type of variables recorded (e.g. correct button press, response time) and what statistics were used to establish that the subjects were performing the task as expected (e.g. mean, range, and/or standard deviation across subjects).*

Acquisition

Imaging type(s)	*Specify: functional, structural, diffusion, perfusion.*
Field strength	*Specify in Tesla*
Sequence & imaging parameters	*Specify the pulse sequence type (gradient echo, spin echo, etc.), imaging type (EPI, spiral, etc.), field of view, matrix size, slice thickness, orientation and TE/TR/flip angle.*
Area of acquisition	*State whether a whole brain scan was used OR define the area of acquisition, describing how the region was determined.*

Diffusion MRI ☐ Used ☐ Not used

Preprocessing

Preprocessing software	*Provide detail on software version and revision number and on specific parameters (model/functions, brain extraction, segmentation, smoothing kernel size, etc.).*
Normalization	*If data were normalized/standardized, describe the approach(es): specify linear or non-linear and define image types used for transformation OR indicate that data were not normalized and explain rationale for lack of normalization.*
Normalization template	*Describe the template used for normalization/transformation, specifying subject space or group standardized space (e.g. original Talairach, MNI305, ICBM152) OR indicate that the data were not normalized.*

Noise and artifact removal	*Describe your procedure(s) for artifact and structured noise removal, specifying motion parameters, tissue signals and physiological signals (heart rate, respiration).*
Volume censoring	*Define your software and/or method and criteria for volume censoring, and state the extent of such censoring.*

Statistical modeling & inference

Model type and settings	*Specify type (mass univariate, multivariate, RSA, predictive, etc.) and describe essential details of the model at the first and second levels (e.g. fixed, random or mixed effects; drift or auto-correlation).*
Effect(s) tested	*Define precise effect in terms of the task or stimulus conditions instead of psychological concepts and indicate whether ANOVA or factorial designs were used.*

Specify type of analysis: ☐ Whole brain ☐ ROI-based ☐ Both

Statistic type for inference (See Eklund et al. 2016)	*Specify voxel-wise or cluster-wise and report all relevant parameters for cluster-wise methods.*
Correction	*Describe the type of correction and how it is obtained for multiple comparisons (e.g. FWE, FDR, permutation or Monte Carlo).*

Models & analysis

n/a	Involved in the study
☐	☐ Functional and/or effective connectivity
☐	☐ Graph analysis
☐	☐ Multivariate modeling or predictive analysis

Functional and/or effective connectivity	*Report the measures of dependence used and the model details (e.g. Pearson correlation, partial correlation, mutual information).*
Graph analysis	*Report the dependent variable and connectivity measure, specifying weighted graph or binarized graph, subject- or group-level, and the global and/or node summaries used (e.g. clustering coefficient, efficiency, etc.).*
Multivariate modeling and predictive analysis	*Specify independent variables, features extraction and dimension reduction, model, training and evaluation metrics.*
